# Use of Corticosteroids in Prenatal Treatment of Congenital Pulmonary Adenomatoid Malformation: Integrative Review

**DOI:** 10.1055/s-0041-1741517

**Published:** 2022-02-02

**Authors:** Isadora Macedo Lins Parentes Fortes, João Renato Bennini Junior

**Affiliations:** 1 Fetal Medicine Program, Department of Gynecology and Obstetrics, Women's Hospital, University of Campinas, Campinas, SP, Brazil

**Keywords:** congenital pulmonary adenomatoid malformation, fetal treatment, corticosteroid, betamethasone, hydrops, malformação adenomatoide pulmonar congenital, tratamento fetal, corticosteroide, betametasona, hidropsia

## Abstract

**Objective** To review data on the use of corticosteroids for the treatment of fetuses with high-risk congenital pulmonary adenomatoid malformation (CPAM).

**Methods** Integrative review based on the literature available on MEDLINE and LILACS, including articles published until November, 2020.

**Results** The initial search resulted in 87 articles, 4 of which were selected for analysis, with all of them being retrospective descriptive observational studies. In the group of fetuses that received only a single corticosteroid cycle, the hydrops resolution rate was 70%, and the survival rate was 83.8%. In fetuses treated with 2 or more cycles of corticosteroids, there was an improvement in the condition of hydrops or edema in a single body compartment in 47%, and survival of 81.8% of the fetuses.

**Conclusion** The use of corticosteroids for the prenatal treatment of high-risk CPAM appears to be associated with an improvement in perinatal outcomes.

## Resumo

**Objetivo** Revisar os dados sobre o uso de corticoide no tratamento de fetos com malformação adenomatoide pulmonar congênita (MAPC) de alto risco.

**Métodos** Revisão integrativa com base na literatura disponível no MEDLINE e LILACS, incluindo artigos publicados até novembro de 2020.

**Resultados** A busca inicial resultou em 87 artigos, dos quais 4 foram selecionados para análise, todos tratando-se de estudos observacionais descritivos retrospectivos. No grupo de fetos que recebeu apenas um único ciclo de corticosteroide, a taxa de resolução da hidropsia foi de 70% e a taxa de sobrevida de 83,8%. Em fetos tratados com 2 ou mais ciclos de corticosteroides, houve melhora do quadro de hidropsia ou edema em um único compartimento corporal em 47% dos fetos e taxa de sobrevida de 81,8%.

**Conclusão** O uso de corticosteroides para o tratamento pré-natal da MAPC de alto risco parece estar associado à melhora dos resultados perinatais.

## Introduction

Congenital pulmonary adenomatoid malformation (CPAM) consists of a relatively rare defect in the lung development and airways. 1 It is generally characterized as a benign hamartoma that occurs due to anomalous ramifications of the bronchioles during pulmonary morphogenesis between 7 and 15 weeks of gestation. 2 3 This abnormal growth of the bronchioles results in a suppression of alveolar growth and the consequent formation of multicystic masses that start to replace the healthy lung structure. 4 The possible cellular mechanisms involved in the development of these malformations are unknown. However, histological assessments show an increase in cell proliferation and a marked decrease in apoptosis compared with normal fetal lung tissue. 5

The lesions are usually unilateral and may occupy the entire fetal hemithorax. In only 15% of cases, bilateral involvement of the chest is observed. The vascular supply comes from the pulmonary vessels, and there may be communication between the lesion and the adjacent normal lung tissue. 4 The Stocker et al. 6 classification, performed in 1977, divides CPAM into 3 distinct histopathological subtypes: type 1, which is the most common and consists of large cysts (2–10 cm in diameter); type 2, which is characterized by multiple small cysts (0.5–2 cm) and solid areas, in addition to being more associated with other fetal anomalies; and type 3, which consists of multiple microscopic cysts (< 0.5 cm in diameter) giving the lesion a solid appearance. 6 Later, Stocker et al. 6 and other authors described two additional subtypes seen less frequently (types 0 and 4). 7 This histological classification into 5 subtypes has been replaced by a simpler one elaborated by Adzick et al. in 1985. 8 This classification divided the lesions based on their appearance into macrocystic (≥ 5 cm) and microcystic (< 5 cm). 8

Fetal diagnosis is usually performed by obstetric ultrasound, but nuclear magnetic resonance can also be used. Both are important for identifying the location of the pulmonary lesion and characterizing its appearance, assessing the blood supply, and determining the occurrence of changes in the positions of the thoracic structures. 1 Ultrasonographic evaluation also allows the identification of the presence of polyhydramnios and hydrops, in addition to making differential diagnoses. 2

Through ultrasonography, it is possible to quantify the volume of the lesion, which is an important prognostic indicator. Lesions are defined as high-risk when the CPAM volume ratio (CVR) is greater than 1.6. In these cases, there is an increased risk of developing hydrops, which is a predictor of impending fetal death with mortality of up to 100%. 9 10 Another factor related to a worse prognosis are lesions with a significant macrocystic component, whose growth and expansion are unpredictable. 9

Prenatal interventions are performed taking into account factors such as gestational age, size of the thoracic lesion, presence or absence of fetal hydrops and maternal health. 8 In hydropic fetuses with less than 32 weeks of gestation without the presence of a dominant cyst, open fetal surgery is indicated. If hydropic fetuses have one or more macrocysts, the conduct is to perform a thoracoamniotic shunt. 2 As an alternative to invasive treatments, empirical maternal administration of betamethasone has also been used with a possibility of treatment, associated with hydrops resolution and increased survival. 11

In view that the best treatment for fetuses diagnosed with high-risk CPAM still remains controversial, and the use of corticosteroids has shown favorable outcomes, 11 it is opportune to conduct this integrative review to assess the possible benefits of using this non-invasive therapy. The aim of the present study was to conduct a review of the published evidence on the use of corticosteroids for the treatment of fetuses with high-risk CPAM to assess a possible improvement in perinatal results.

## Methods

### Guiding Question

Does the use of corticosteroids in the treatment of CPAM improve perinatal outcomes?

### Literature Search

This integrative review was performed through research conducted in the Online Medical Literature Analysis and Recovery System (MEDLINE), PubMed, and Latin American and Caribbean Health Sciences Literature (LILACS). The descriptors used in the English language were the following Mesh terms: *fetal therapies* AND *congenital adenomatoid lung malformation* . In this research, there was no restriction on any year of publication and articles published up to November 2020 were included. This research was complemented by reading references of the articles found and by additional automated search, using the search for related articles in PubMed.

### Data Evaluation

For the structure of the integrative review, the methodology described by Souza et al. 12 was followed. The inclusion criteria defined for the selection of articles were articles published in Portuguese, English, and Spanish as well as articles in full that reported the theme related to the integrative review. Studies that reported cases of CPAM, but without interventions or surgical interventions with a choice of treatment were excluded. The review included cases of fetuses diagnosed with high-risk CPAM whose intrauterine treatment involved the use of corticosteroids. High-risk fetuses were considered to be those with the following findings: hydrops and/or CVR > 1.6. Two analyses were undertaken: first, an initial grouping of all available data was performed and, subsequently, a sub-analysis of the studies with better quality evidence, which allowed a better determination of the results of the use of antenatal corticotherapy for the treatment of CPAM. All studies were compared to ensure there were no duplicates or overlapping samples.

### Data Analysis

The analysis and synthesis of the data extracted from the articles were performed in a descriptive manner, making it possible to analyze, quantify, describe, and classify the data, to gather the knowledge produced on the theme explored in this integrative review. The data were consolidated into the following categories: dosage and dose of corticoid used, number of corticosteroid cycles, presence, or absence of hydrops, average CVR, and average gestational age at the start of treatment, hydrops regression rates, reduced CVR, survival of fetuses, and mortality after treatment.

## Results

**Fig. 1:**
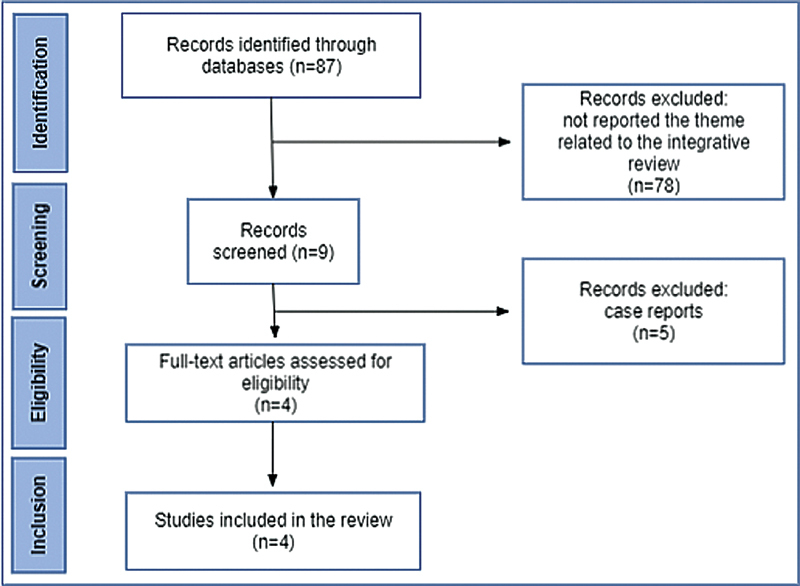
Summary of study selection.

The initial research identified a total of 87 articles. After analysis by title and abstract, 78 papers were excluded. Of the remaining nine studies, four were selected after reading the full text, all of which were descriptive retrospective observational studies ( Fig. 1 ). The 4 selected studies comprise fetal results regarding treatment with corticosteroids during the prenatal period performed on 79 fetuses diagnosed with high-risk CPAM. 13 14 15 16 Chart 1 shows a description of the selected articles and the main results reported by them.

**Chart 1:** Main information of the studies of fetuses with congenital high-risk pulmonary adenomatoid malformation treated with maternal corticosteroid administration

Article	Type of article	Study objective	Place of study	Studied population	Number of individuals	Main results
Morris et al. 13	Retrospective review	Review the experience with CPAMs to determine the fetal response to steroid therapy	Cincinnati, OH, USA	Fetuses diagnosed with high-risk CPAM and treated with at least one course of betamethasone	44 with CPAM, of which 15 were selected to receive at least one dose of corticosteroids	● Thirteen were hydropic and 2 were nonhydropic. ● Seven of the 13 hydropic fetuses (54%) shown an initial response to steroid administration, whereas the 2 nonhydropic high-risk fetuses progressed to birth without developing hydrops. ● Seven of 15 patients, however, resulted in the fetal demise or early postnatal death, giving a survival rate of 53%.
Curran et al. 14	Retrospective review	Evaluate the effect of prenatal steroid treatment in fetuses with sonographically diagnosed CPAM	San Francisco, CA, USA	Patients with a diagnosis of fetal CPAM with a predominately microcystic lesion and not submitted to a fetal surgical intervention for CPA and who received maternal administration of a single course of prenatal steroids	Sixteen pregnancies with predominately microcystic CPAM treated with a single course of prenatal steroids (3 were excluded because of lack of follow-up information.	● All 13 fetuses survived to delivery and 11 survived to neonatal discharge. ● At the time of steroid administration, all patients had CVR greater than 1,6, and 9 of them also had nonimmune hydrops fetalis. ● After a course of steroids, CVR decreased in 8 of the 13 patients, and hydrops resolved in 7 of the 9 patients with hydrops. ● The 2 patients whose hydrops did not resolve with steroid treatment did not survive to discharge.
Peranteau et al. 15	Retrospective review	Present the experience with the management fetuses with large congenital lung lesions receiving multiple courses of maternal BMZ and provide an update on the response of those receiving a single course.	Philadelphia, PA, USA	Fetuses with congenital lung lesions managed with maternal BMZ	Forty-three patients were managed with prenatal steroids (28 with single course and 15 with multiple courses)	● Single course recipients demonstrated a reduction in lesion size and resolution of hydrops in 82% and 88% of patients respectively compared with 47% and 56% in recipients of multiple steroid courses. ● Survival of multiple course patients (86%) was comparable to that of single course patients (93%) and improved compared with non-treated historical controls. ● Multiple course recipients demonstrated an increased need for open fetal surgery and postnatal surgery at a younger age.
Derderian et al. 16	Retrospective review	Review treatment approaches and outcomes of fetuses who received multiple courses of maternal betamethasone at two tertiary fetal treatment centers.	Cincinnati, OH, and San Francisco, CA, USA	Patients treated with multiple courses of prenatal steroids for high-risk CPAMs	Eight patients with high-risk CPAM	● All patients (9) but one either had an increased CVR or number of fluid-containing compartments involved after a single course of antenatal betamethasone, prompting additional courses. ● Four patients stabilized, three improved and two progressed after the second course. ● The two cases with disease progression underwent an in utero resection. ● There were one in utero fetal demise and two deaths within the delivery room. Both fetuses that underwent a fetal resection died. ● All but one mother who delivered a viable fetus had complications of pregnancy.

Abbreviations: BMZ, betamethasone; CPAM, congenital pulmonary adenomatoid malformation; CVR, CPAM volume ratio.

Two studies evaluated fetuses that were treated only with a single course of corticosteroids, and two others evaluated those whose treatment involved the use of one or multiple courses of corticosteroids. The treatment was performed with maternal administration of betamethasone intramuscularly in a total dose of 12 to 12.5 mg divided into 2 applications with an interval of 24 hours between them.

Morris et al. 13 evaluated a total of 15 fetuses treated with a single corticosteroid cycle. Of these, 13 were hydropic and had an average CVR of 2.82 ± 1.63. The other two fetuses were non-hydropic and had an average CVR of 1.82 ± 0.08. The mean gestational age at the time of application of the corticoid cycle was 24 ± 4.1 weeks. Regarding the type of lesion evaluated, seven fetuses had macrocystic lesions, seven of them microcystic lesions, and only one had hybrid lesions. After the corticosteroid treatment was performed, hydrops was resolved in 7 (54%) of the 13 fetuses, 3 of whom required additional therapeutic interventions: 2 of them had macrocystic lesions, having undergone thoracoamniotic shunt, and 1 fetus was subjected to open fetal surgery, evolving to complete resolution of the hydrops. It was observed that the two non-hydropic fetuses remained stable without progression to hydrops. The survival rate was 53% ( *n* = 8). Six fetuses died, due to non-responsiveness to corticosteroid treatments and surgical procedures, emergency delivery due to placental abruption and preeclampsia, and request for termination of pregnancy by parents.

The study by Curran et al. 14 included an evaluation of 13 fetuses with CPAM and predominantly microcystic lesion treated with only one corticosteroid cycle. Among the fetuses, the presence of hydrops was observed in 9 of them, only the presence of ascites in 2 fetuses, and in another 2, a CVR greater than 1.6. At the time of application of the corticoid cycle, the mean gestational age was 24.55 ± 1.53 weeks. After treatment, hydrops was completely resolved in 77.8% of hydropic fetuses and ascites was resolved in the 2 fetuses. The average interval between the treatment and hydrops resolution was 29.9 days. The study also showed that all fetuses showed cardiac displacement and diaphragmatic eversion due to the presence of a large lesion in the chest cavity. The average CVR at diagnosis was 2.72 ± 0.92. With the completion of the betamethasone cycle, it was observed that the growth of the lesion slowed in 61.5% of the cases, with an average decrease in the CVR values of 1.6 ± 1.1, in addition to an improvement in the mediastinal displacement in 100% of the cases. In three of the eight patients, the lesions regressed completely and were no longer seen on ultrasound. All 13 fetuses survived delivery, with an average gestational age at delivery of 35.6 weeks, and 84.6% of them survived neonatal discharge.

Among the two studies that evaluated fetuses treated with one or more cycles of corticosteroids, one performed a retrospective review of fetuses with congenital lung lesions, which included macro and microcystic CPAM ( *n* = 31), hybrid lesions ( *n* = 6), bronchial atresia ( *n* = 3), congenital lobar emphysema ( *n* = 1), and pulmonary sequestration ( *n* = 2). 15 The study evaluated 43 treated fetuses: 28 of whom received only 1 cycle, 13 of them received 2 cycles, and 2 were treated with 3 cycles. In this study, it is not clear which criteria were used to determine whether to perform the treatment with a single or multiple doses. The average gestational age of the fetuses at the time they received the 1 ^st^ , 2 ^nd^ , and 3 ^rd^ cycles was 23.4 ± 2.2, 24 ± 2 and 25.3 ± 0.3, respectively. The mean CVR, in turn, was 2 ± 0.6, 2.6 ± 0.7 and 2.5 ± 0.4, respectively. Of the treated fetuses, 42% had a diagnosis of hydrops (8 from the group that received a single course, and 10 from those that received multiple courses). The presence of mediastinal deviation was evidenced in 100% of the patients. As a result of the treatment, hydrops was resolved in 71% of cases, 88% in the single cycle group, and 56% in the multiple cycle group, in addition to an improvement in mediastinal deviation in 51% of the fetuses (16/28 of those who received a single course and in 6/15 of those who received multiple courses). Regarding the decrease in the CVR value, a reduction was observed in all 2 groups, with 82% in the single cycle and 47% in the multiple cycle. It was shown that 93% ( *n* = 26) of patients who received a single course and 86% ( *n* = 12) of patients who received multiple courses survived until hospital discharge. Among the four deaths observed, one occurred due to interruption of the pregnancy requested by the parents, and the others due to respiratory failure, extreme prematurity, and fetal death.

Derderian et al. 16 performed the evaluation of eight fetuses with CPAM of a predominantly microcystic component treated with two cycles of betamethasone. The study reports that the criterion used to perform the second dose of corticosteroid was persistence of hydrops and/or maintenance of high CVR values. Hydrops was evident in five cases, while three cases had fluid in only one compartment (ascites). The average gestational age when administering the 1 ^st^ course of prenatal betamethasone was 22 weeks. The progression of the disease was observed in all but one case, thus leading to the application of a 2 ^nd^ course of steroids, which was administered at an average gestational age of 25 weeks. In the fetus with improved hydrops, a second cycle was performed because the CVR remained persistently elevated. Following the second corticosteroid cycle, ultrasound findings showed improvement in three cases, stabilization in three other cases, and progression in two cases. Regarding CVR, the mean value at diagnosis was 2.6. With the initial treatment, CVR stabilization was observed in five patients, and progression in three of them. After the second cycle, two fetuses showed a reduction in CVR, while the remaining four showed stabilization of the value, and two showed an increase. The disease progressed in two fetuses, despite a second course of maternal betamethasone. Both died, with one being an intrauterine fetal death and the other in the immediate postnatal period. The average gestational age at delivery was 34 weeks.

Charts 2 and 3 show a description of the selected articles and the fetal results reported in the articles.

**Chart 2:** Data from studies of fetuses with congenital high-risk pulmonary adenomatoid malformation treated with maternal corticosteroid administration

Article	Number of fetuses treated	Corticosteroid used*	Number of cycles	Type of lung injury	Fetuses with hydrops	Fetuses without hydrops	Mediastinal shift	Average gestational age (weeks)	Average CVR before treatment
Morris et al. 13	15	Betamethasone 12.5 mg 02 doses	1	Macrocystic ( *n* = 7) Microcystic ( *n* = 7) Hybrid ( *n* = 1)	13	2	NA	24 ± 4.1	2.62 ± 1.51
Curran et al. 14	13	Betamethasone 12 mg 02 doses	1	Predominantly microcystic	9	2 with ascites 2 without edema	100%	24.55 ± 1.53	2.72 ± 0.92
Peranteau et al. 15	43 #	Betamethasone 12 mg 02 doses	1	Macrocystic and microcystic	18	25	43	23.4 ± 2.2	2 ± 0.6
			2	Macrocystic and microcystic				24 ± 2	2.6 ± 0.7
			3	Macrocystic and microcystic				25.3 ± 0.3	2.5 ± 0.4
Derderian et al. 16	8	Betamethasone 12.5 mg 02 doses	2	Predominantly microcystic	5	3 with ascites	NA	First cycle: 22 Second cycle: 25	2.8

Abbreviations: CVR, CPAM volume ratio ; NA, not analyzed.

* Intramuscular use with an interval of 24 hours between doses.

# Included for treatment and data analysis fetuses with congenital lung injuries (CPAM = 31, hybrid injuries = 6, pulmonary sequestration = 2, bronchial atresia = 3, congenital lobar emphysema = 1).

**Chart 3:** Data from studies of fetuses with congenital high-risk pulmonary adenomatoid malformation treated with maternal corticosteroid administration

Article	Regression of hydrops	Interval between treatment and resolution of hydrops	Regression of CVR	Resolution of mediastinal shift	Survival rate	Mean gestational age of childbirth (weeks)
Morris et al. 13	54% a	NA	NA	NA	53%	NA
Curran et al. 14	77.8% of hydrops 100% of those with ascites	29.9 days	61.5%	100%	100% b	35.6
Peranteau et al. 15	Single cycle: 88% Multiples cycles: 56% General: 71%	NA	Single cycle: 82% Multiples cycles: 47%	Single cycle: 57% Multiples cycles: 40% General: 51%	Single cycle: 93% Multiples cycles: 86% General: 98%	NA
Derderian et al. 16	First cycle: 12.8% with improvement	NA	First cycle: 62.5% with stabilization	NA	75%	34
	Second cycle: 37.5% with improvement 37.5% with stabilization		Second cycle: 25% with improvement 50% with stabilization			

Abbreviations: CVR, CPAM volume ratio; NA, not analyzed.

a 3 fetuses needed complementary surgery after corticosteroid therapy.

b 84.6% survived hospital discharge.

## Discussion

The use of corticosteroids for the treatment of congenital high-risk pulmonary adenomatoid malformation is a recent therapy. Its use as a non-invasive therapy option is associated with improvement or regression of hydrops and reduced CVR, in addition to reduced mortality rate. Studies show that, without treatment of fetuses with high-risk CPAM, the risk of developing hydrops was increased, which is an important predictor of fetal mortality, reaching up to 100%. 9 10 Thus, the data available in the literature show that its use has significantly contributed to the improvement of fetal and perinatal prognosis.

The analysis of the data present in the articles included in this integrative review shows that there was a better rate of hydrops resolution in the group of fetuses treated with only one course of steroids (70%) compared with fetuses that received two or more courses of steroids (47%). 13 14 15 16 Regarding the survival rate, there was no great difference between the two groups, with the survival rate of fetuses being 83.3% in the group treated with a cycle, and 81.8% in the group that received multiple cycles of corticosteroids. 13 14 15 16 It is of great importance that the criteria for determining the use of treatment with single or multiple courses of corticosteroids are better defined, since the outcome in the resolution of hydrops can be influenced.

One of the studies compared responders and non-responders to treatment, and it was observed that there was no significant difference in the mean gestational age of administration of corticosteroids (23.12 ± 3.71 vs 25.24 ± 4.82) or CVR measurement (2.29 ± 0.69 vs 3.03 ± 1.95). There was also no significant difference in the macro or microcystic CPAM in relation to its response to corticosteroids. 13 Therefore, there does not seem to be any difference in relation to the gestational age at which corticosteroids are administered by the mother, nor is there any change in the effectiveness of the treatment according to the type of lesion.

Derderian et al. 16 inferred that six of the seven patients (excluding the case that resulted in intrauterine fetal death) had a pregnancy complication, such as mirror syndrome, premature rupture of membranes, polyhydramnios, premature labor, or chorioamnionitis. The study concluded that the high rate of prematurity and other complications of pregnancy suggest that the edematous state present in high-risk CPAM is a risk factor for complications in pregnancy. 14 It is important, thus, that special attention be paid to the occurrence of possible gestational complications during treatment with corticosteroids.

The research performed showed that the studies undertaken so far are uniform in terms of the criteria for indicating the treatment and the dosage for the use of betamethasone. However, they are quite scarce and heterogeneous with regards to defining the number of cycles that should be performed and the time interval between cycles, in addition to being retrospective evaluations that did not perform the comparison between groups with and without the use of corticosteroids for better proof of the beneficial effects of its use.

The results obtained in this research can be used as a basis for the elaboration of care protocols, in addition to research projects aiming at the development of studies with higher quality of evidence, since the current data are limited and heterogeneous.

The limitations of the review method performed consisted of the use of retrospective studies, which did not allow for comparisons between study groups, in addition to the fact that the research was not performed in all languages or in all databases.

## Conclusion

Despite the data found in the literature showing favorable outcomes with the use of corticotherapy in the treatment of fetuses with high-risk CPAM, it is still necessary to carry out further studies with high evidence quality on the subject so that more accurate information can be obtained.
